# The frequency of HLA alleles in a population of Inuit women of northern Quebec

**DOI:** 10.3402/ijch.v72i0.21350

**Published:** 2013-08-05

**Authors:** Stephanie Metcalfe, Michel Roger, Marie-Claude Faucher, François Coutlée, Eduardo L. Franco, Paul Brassard

**Affiliations:** 1 Department of Epidemiology, Biostatistics and Occupational Health, McGill University, Montreal, Quebec, Canada; 2 Department of Microbiology, Centre Hospitalier de l'Université de Montréal, Montreal, Quebec, Canada; 3 Department of Oncology, McGill University, Montreal, Quebec, Canada; 4 Department of Medicine, McGill University, Montreal, Quebec, Canada

**Keywords:** human leukocyte antigen, Inuit, Nunavik, Canada

## Abstract

**Background:**

Human leukocyte antigen (HLA) alleles code for proteins that are involved in the recognition of foreign antigens and activation of the immune system. The frequency of HLA alleles varies across different populations.

**Objective:**

To describe the frequency of HLA alleles in a population of Inuit women of Nunavik, Quebec, Canada.

**Design:**

A cohort of women was recruited from 4 different communities between January 2002 and December 2007. HLA-B*07, HLA-DQB1*03, DQB1*06:02, DRB1*13 and DRB1*15:01 alleles were typed by PCR sequence-specific primers (PCR-SSP) and HLA-E and G alleles were type by DNA-sequencing procedures.

**Results:**

We obtained data on 524 participants. The most frequent HLA alleles in this population were HLA-E*01:03, HLA-G*01:04:01 and HLA-DQB1*03, and they were found in 89, 75 and 94% of the population, respectively.

**Conclusions:**

The distribution of HLA alleles in Nunavik, Quebec is unique when compared to other populations in Canada or around the world.

Nunavik is the arctic and sub-arctic region of Northern Quebec, Canada. For 4,000 years this region has been inhabited by nomads, the first residents of Nunavik travelled from Asia to North America via the Bering Strait. The Inuit people are descendants of the Thule, who appeared in the western part of Northern Canada around 1,000 AD. The earliest archaeological evidence of the Thule people in Nunavik dates back to around the thirteenth century (1,2). Today, Nunavik's population of 11,000 lives in 14 communities along the coast of Hudson Bay, Hudson Straight and Ungava Bay. Ninety percent of the population of Nunavik self-identifies as Inuit. This population is not only geographically isolated but has a unique cultural and socio-demographic make-up (1,3) (Fig. 1).

**Fig. 1:**
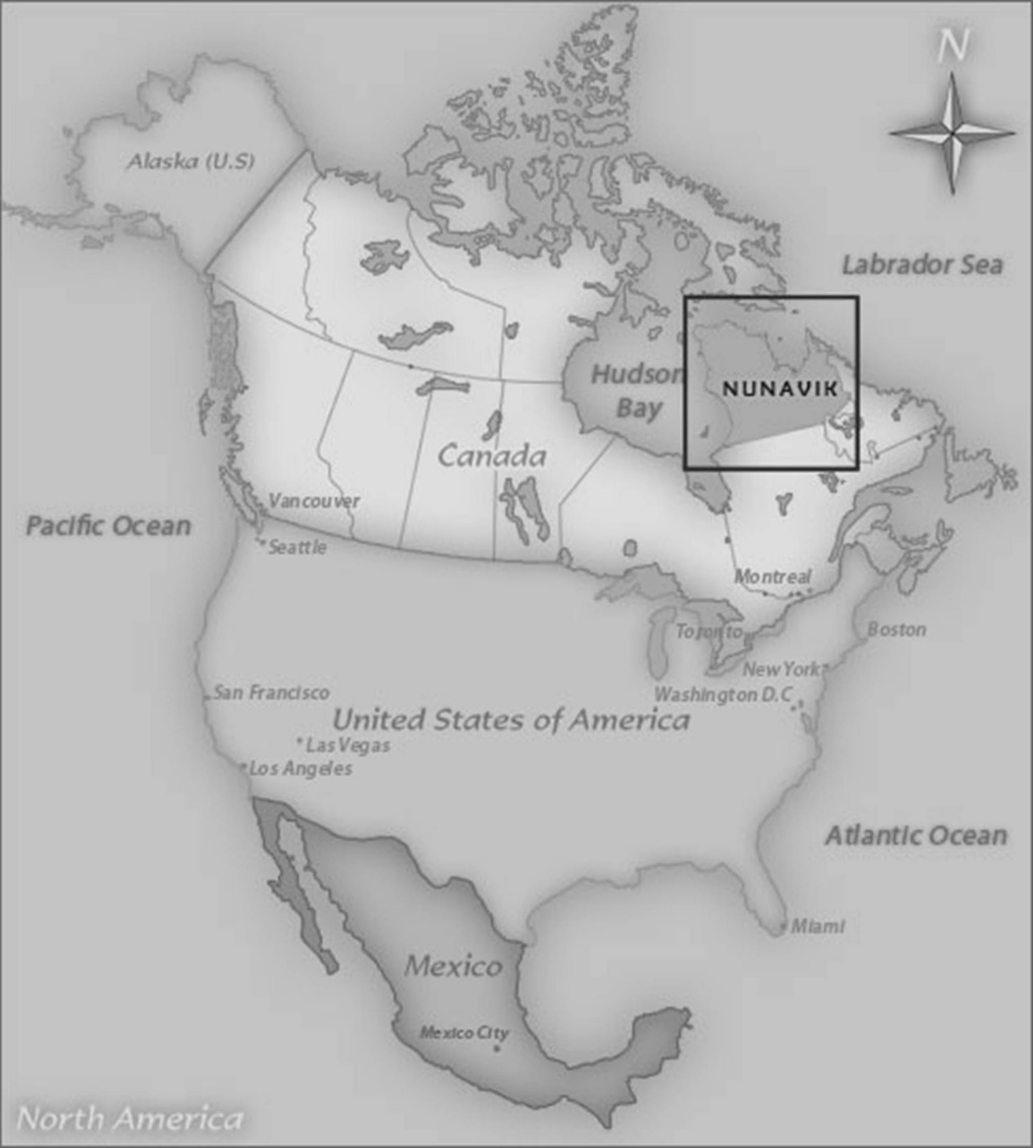
The location of Nunavik, Quebec in North America.

Human leukocyte antigens (HLA) are part of the major histocompatibility complex (MHC) in humans. The HLA family of genes code for cell surface proteins, which present antigens (foreign or self) to immune cells, and other proteins involved in internal processing of antigens (4–6). HLA class I proteins include HLA-A, B, C, E, F and G and are expressed on all nucleated cells. Class II proteins include HLA-DP, DQ and DR and are expressed on immune cells (lymphocytes and macrophages) (4).

HLA genes are highly polymorphic allowing HLA proteins to bind to and present a variety of antigenic proteins to immune cells (4). Determining which HLA alleles are present in a population (and their frequencies) could provide insights into the origin of Native American groups, the evolution of HLA polymorphisms (a point in a DNA sequence where there is variation), and the population's genetic susceptibility to disease. Polymorphisms in various HLA genes have been linked with susceptibility to viral infections such as HPV, HIV, HBV and HCV (4–8). It is hypothesised that individuals with HLA polymorphisms with a higher binding affinity to a particular viral antigen are better at clearing the infection.

An allele is an alternative form or version of a gene. An individual's genotype is the 2 alleles inherited for a particular gene. If an individual is homozygous for a gene, both their alleles are the same and if they are heterozygous they have 2 different versions of the allele. A haplotype is a set of DNA polymorphisms along a chromosome that tend to be inherited together (9). HLA alleles are named in this way: HLA–locus name*allele number. The first 2 numbers after the asterisk (*) are the allele number and any numbers after that are the allele subtype (10). For example HLA-G*01:04:01, 01 is the allele number and 04:01 is the allele subtype.

## Methods

The data used for this analysis were taken from a prospective cohort of Inuit women used to study the prevalence, incidence and determinants of human papillomavirus (HPV) infection. Details of this study have been previously published (11). Nurse practitioners systematically asked women aged 15–69 presenting for a regularly scheduled Pap test at a clinic in one of the 4 participating communities between January 2002 and December 2007 if they wanted to participate. All eligible women provided written consent, and ethics approval was obtained from the McGill Institutional Review Board and the Tulattavik Health Center.

At enrolment, cervical specimens were collected and used for HLA typing. Low-resolution typing was used to determine the presence of HLA-B*07 and 4 class II alleles: HLA-DQB1*03, DQB1*06:02, DRB1*13 and DRB1*15:01 (these alleles were associated with cervical cancer in previous literature and were chosen a priori). They were typed using a PCR technique that used sequence-specific primers (PCR-SSP), according to a well-established procedure where pairs of oligonucleotides were used as primers for amplification of the HLA alleles (12,13). Class I HLA-E and G alleles were typed using high-resolution DNA typing; therefore, all of the alleles present in the population were captured. HLA-G and HLA-E alleles were determined through direct DNA sequencing of the nucleotide regions encompassing the HLA-E exon 3 (218bp) and HLA-G exons 2–4 (1718bp) using purified DNA from cervical samples (14).

## Results

The study population comprised 524 women. The most common class I and II alleles were HLA-E*01:03 and HLA-DQB1*03 which were found in 89.3 and 94.2% of women, respectively. HLA-B*07, DQB1*06:02, DRB1*13 and DRB1*15:01 were all found in <5% of the women in this population (Table I). Table II shows the HLA haplotypes of the 5 alleles that were chosen a priori (HLA-B*07, DQB1*03, DQB1*06:02, DRB1*13, DRB1*15:01). None of these haplotypes were present in more than 5% of the population.

**Table I:** The frequency of HLA alleles, both the frequency of women with the allele (N=524) and for HLA-G and E the frequency of alleles in the population (N=1048)

HLA allele	Frequency of women with HLA allele			Frequency of the HLA alleles in the population		
	
	n	N*	%	n	N	%
HLA-B*07	23	514	4.47			
HLA-E*01:01	293	524	55.92	343	1048	32.73
HLA-E*01:03	468	524	89.31	693	1048	66.13
HLA-G*01:01:01	278	524	52.86	328	1048	31.30
HLA-G*01:01:02	146	524	28.82	159	1048	15.17
HLA-G*01:01:03	11	524	2.10	11	1048	1.05
HLA-G*01:01:08	4	524	0.76	4	1048	0.38
HLA-G*01:03	4	524	0.76	4	1048	0.38
HLA-G*01:04:01	399	524	75.38	526	1048	50.19
HLA-G*01:06	13	524	2.48	14	1048	1.34
HLA-G*01:07	1	524	0.19	1	1048	0.10
HLA-DQB1*03	486	516	94.19			
HLA-DQB1*06:02	19	523	3.63			
HLA-DRB1*13	25	532	4.70			
HLA-DRB1*15:01	20	527	3.80			

* The denominator is not always consistent because some of the specimens could not be tested by PCR.

**Table II:** The frequency of women with HLA haplotypes (N=524)

HLA haplotype	n	N*	%
B*07-DQB1*03	18	494	3.64
B*07-DQB1*06:02	8	479	1.67
B*07-DRB1*13	1	510	0.20
B*07-DRB1*15:01	8	502	1.59
DQB1*03-DQB1*06:02	13	505	2.57
DQB1*03-DRB1*13	15	511	2.94
DQB1*03-DRB1*15:01	14	507	2.76
DQB1*06:02-DRB1*13	1	517	0.19
DRB1*15:01-DQB1*06:02	19	511	3.72
DRB1*15:01-DRB1*13	1	520	0.19
B*07-DRB1*15:01-DQB1*06:02	8	487	1.64

* The denominator is not always consistent because some of the specimens could not be amplified.

Two HLA-E alleles and 8 HLA-G alleles were found in the population of Nunavik. HLA-E*01:01, the wild type allele, made up 32.7% of the alleles in the population and HLA-E*01:03 made up 66.2%. The most common HLA-G alleles were: HLA-G*01:04:01 (50.4%), G*01:01:01 (31.4%) and G*01:01:02 (15.2%) (Table I). A total of 47.9% of the HLA-G alleles in this population were from the wild type HLA-G*01:01 lineage (includes HLA-G*01:01:01, G*01:01:02, G*01:01:03 and G*01:01:08). Table III shows the frequencies of the genotypes of HLA-G*01:01:01, G*01:01:02 and G*01:04:01.

**Table III:** The frequency of the homozygous and heterozygous genotypes for the 3 most common HLA-G alleles (N=524)

HLA-genotype	n	N	%
	G*01:01:01		
Homozygous	51	524	9.73
Heterozygous	227	524	43.32
Absent	244	524	46.56
	G*01:01:02		
Homozygous	8	524	1.53
Heterozygous	138	524	26.34
Absent	376	524	71.76
	G*01:04:01		
Homozygous	131	524	25.00
Heterozygous	268	524	51.15
Absent	125	524	23.85

## Discussion

This is the first known study to determine the frequency of HLA alleles in the Inuit of Nunavik, Quebec. A study by Leffell et al. investigated the frequency of HLA alleles in the Yup'ik Inuit population of Alaska in which HLA-B*07:02, DRB1*15:01, DRB1*13:01 and DQB1*06:02 were present in less than 2% of the population and DQB1*03:01 had a frequency 61.7% (15). On the basis of the distribution of these 5 alleles, the Yup'ik population of Alaska and the Inuits of Nunavik are somewhat similar. Unfortunately, the Alaskan study did not type for HLA-E or G.

In women living in Montreal, Quebec, the HLAB*07, HLA-DQB1*06:02, DRB1*13 and DRB1*15:01 alleles were all more frequent than in Nunavik. In 2 Montreal populations, their frequencies were 15, 20, 20 and 23%, respectively (13,16). HLA-DQB1*03 was found in around 60% of the Montreal populations, whereas it was found in almost all of the Inuit women (94%). The reported haplotypes of these 5 alleles were again found in a greater proportion of the Montreal population than the Nunavik population (13,16).

The HLA-G*01:01:01 and HLA-G*01:01:02 alleles were prevalent in Montreal women (45 and 24%), but HLA-G*01:04:01 (which was the most prevalent HLA-G allele in Nunavik, 50%) made up only 9% of the alleles in the population (17). A total of 21, 7 and 2% of the Montreal population had homozygous HLA-G*01:01:01, G*01:01:02 and G*01:04:01 genotypes, respectively, and 46, 34 and 14% had heterozygous G*01:01:01, G*01:01:02 and G*01:04:01 genotypes, respectively (17). The Nunavik Inuit women had a lower percentage of both heterozygote and homozygote HLA-G*01:01:01 and G*01:01:02 genotypes than the Montreal women. The heterozygote and homozygote and G*01:04:01 genotypes were found in 51 and 25% of the Nunavik population, a much greater proportion than in the Montreal population (17).

A study by Matte et al. compiled the frequencies of the HLA-G allele from 10 populations around the world (18). The most common alleles found were HLA-G*01:01:01, G*01:01:02 and G*01:04:01. In comparison, the Nunavik population had lower frequency of HLA-G*01:01:01 and G*01:01:02 and a higher frequency of HLA-G*01:04:01 than the majority of the populations studied by Matte et al. In all populations but one, the most frequent allele was G*01:01:01 (in the German/Croatian population, HLA-G*01:01:02 was the most frequent allele) (18). In the Inuit population of Nunavik, the most common allele was HLA-G*01:04:01 (50%) which makes it unique among these populations.

Limitations to this study include the possibility of regional differences of HLA frequencies within the Nunavik territory and non-random recruitment. In conclusion, the frequency of HLA alleles in Nunavik, Quebec is different from that found in other Canadian populations and other populations around the world. Two alleles in particular, HLA-G*01:04:01 and HLA-DQB1*03 (found in 76 and 94% of individuals, respectively) were found more frequently in the Nunavik population than in other populations which have been studied. Clinical significance of these findings in terms of the potential impact on HPV prevalence or persistence will be subsequently explored. These regional variations could have an impact on disease outcome including not only HPV but also other morbidities such as autoimmune disorders and vaccine efficacy.
